# 1-Methyl-3,3-bis­(phenyl­sulfan­yl)piperidin-2-one

**DOI:** 10.1107/S1600536812021277

**Published:** 2012-05-19

**Authors:** Ignez Caracelli, Paulo R. Olivato, Carlos R. Cerqueira Jr, Jean M. M. Santos, Seik Weng Ng, Edward R. T. Tiekink

**Affiliations:** a BioMat – Departamento de Física, Universidade Federal de São Carlos, 13565-905 São Carlos, SP, Brazil; b Chemistry Institute, Universidade de São Paulo, 05508-000 São Paulo, SP, Brazil; c Department of Chemistry, University of Malaya, 50603 Kuala Lumpur, Malaysia; d Chemistry Department, Faculty of Science, King Abdulaziz University, PO Box 80203 Jeddah, Saudi Arabia

## Abstract

The piperidone ring in the title compound, C_18_H_19_NOS_2_, is in a distorted half-chair conformation, distorted towards a twisted boat, with the central methyl­ene C atom of the propyl backbone lying 0.606 (2) Å out of the plane defined by the other five atoms (r.m.s. deviation = 0.1197 Å). One of the S-bound phenyl rings is almost perpendicular to the least-squares plane through the piperidone ring, whereas the other is splayed [dihedral angles = 75.97 (6) and 44.21 (7)°, respectively]. The most prominent feature of the crystal packing is the formation of helical supra­molecular chains along the *b* axis sustained by C—H⋯O inter­actions. The chains are consolidated into a three-dimensional architecture *via* C—H⋯π inter­actions whereby one S-bound phenyl ring accepts two C—H⋯π contacts.

## Related literature

For background to β-thio-carbonyl compounds, see: Vinhato *et al.* (2011 ▶); Olivato *et al.* (2009 ▶). For related structures, see: Zukerman-Schpector *et al.* (2010 ▶, 2011 ▶). For ring conformational analysis, see: Cremer & Pople (1975 ▶). For the synthesis, see: Zoretic & Soja (1976 ▶).
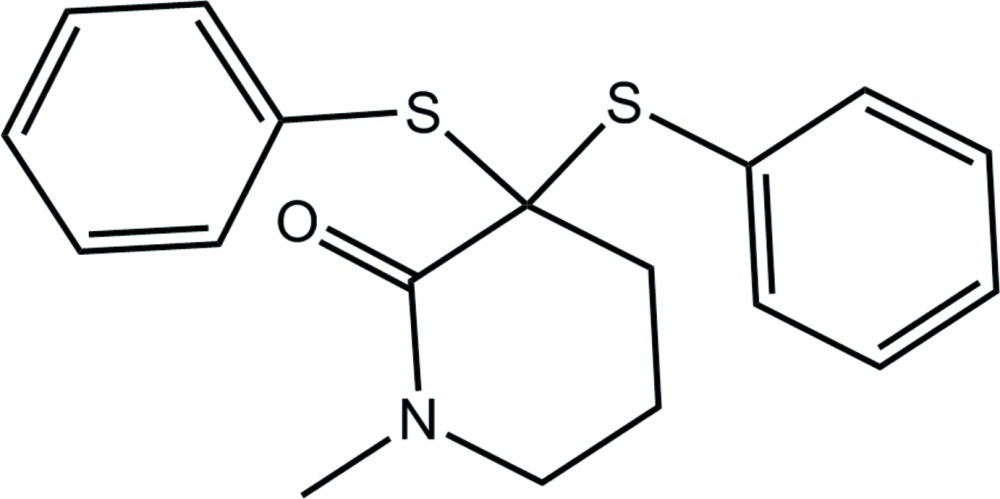


## Experimental

### 

#### Crystal data

C_18_H_19_NOS_2_
*M* *_r_* = 329.48
Orthorhombic, 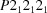

*a* = 8.2103 (1) Å
*b* = 9.8329 (1) Å
*c* = 20.3686 (2) Å
*V* = 1644.38 (3) Å^3^
*Z* = 4
Cu *K*α radiation
μ = 2.93 mm^−1^
*T* = 100 K
0.35 × 0.30 × 0.25 mm

#### Data collection

Agilent SuperNova Dual (Cu at zero) diffractometer with an Atlas detector
Absorption correction: multi-scan (*CrysAlis PRO*; Agilent, 2010 ▶) *T*_min_ = 0.427, *T*_max_ = 0.585
4452 measured reflections
2769 independent reflections
2728 reflections with *I* > 2σ(*I*)
*R*_int_ = 0.016

#### Refinement

*R*[*F* ^2^ > 2σ(*F* ^2^)] = 0.025
*wR*(*F* ^2^) = 0.067
*S* = 1.09
2769 reflections
200 parameters
H-atom parameters constrained
Δρ_max_ = 0.19 e Å^−3^
Δρ_min_ = −0.32 e Å^−3^
Absolute structure: Flack (1983 ▶), 818 Friedel pairs
Flack parameter: 0.024 (14)

### 

Data collection: *CrysAlis PRO* (Agilent, 2010 ▶); cell refinement: *CrysAlis PRO*; data reduction: *CrysAlis PRO*; program(s) used to solve structure: *SIR92* (Altomare *et al.*, 1999 ▶); program(s) used to refine structure: *SHELXL97* (Sheldrick, 2008 ▶); molecular graphics: *ORTEP-3* (Farrugia, 1997 ▶), *DIAMOND* (Brandenburg, 2006 ▶) and *MarvinSketch* (ChemAxon, 2009 ▶); software used to prepare material for publication: *publCIF* (Westrip, 2010 ▶).

## Supplementary Material

Crystal structure: contains datablock(s) global, I. DOI: 10.1107/S1600536812021277/hg5220sup1.cif

Structure factors: contains datablock(s) I. DOI: 10.1107/S1600536812021277/hg5220Isup2.hkl

Supplementary material file. DOI: 10.1107/S1600536812021277/hg5220Isup3.cml

Additional supplementary materials:  crystallographic information; 3D view; checkCIF report

## Figures and Tables

**Table 1:** Hydrogen-bond geometry (Å, °) *Cg*1 is the centroid of the C7–C12 ring.

*D*—H⋯*A*	*D*—H	H⋯*A*	*D*⋯*A*	*D*—H⋯*A*
C11—H11⋯O1^i^	0.95	2.32	3.237 (3)	163
C6—H6b⋯*Cg*1^ii^	0.98	2.95	3.606 (2)	125
C14—H14⋯*Cg*1^iii^	0.95	2.96	3.544 (2)	121

Symmetry codes: (i) 
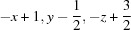
; (ii) 
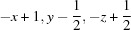
; (iii) 
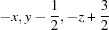
.

**Fig. 1.**
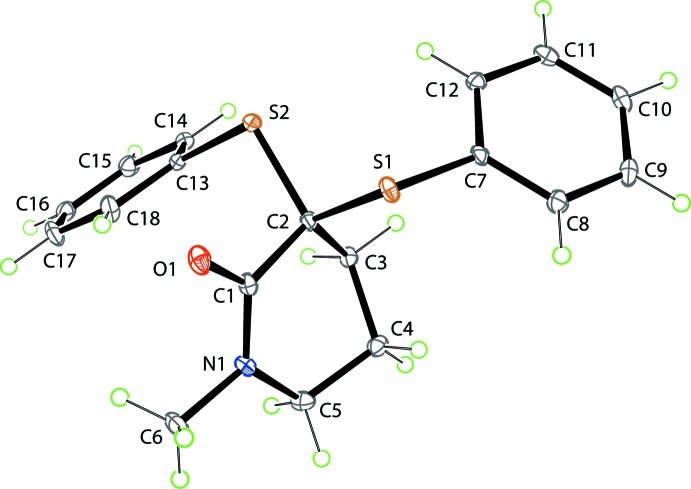
The molecular structure of (I) showing atom labelling scheme and displacement ellipsoids at the 50% probability level (arbitrary spheres for the H atoms).

**Fig. 2.**
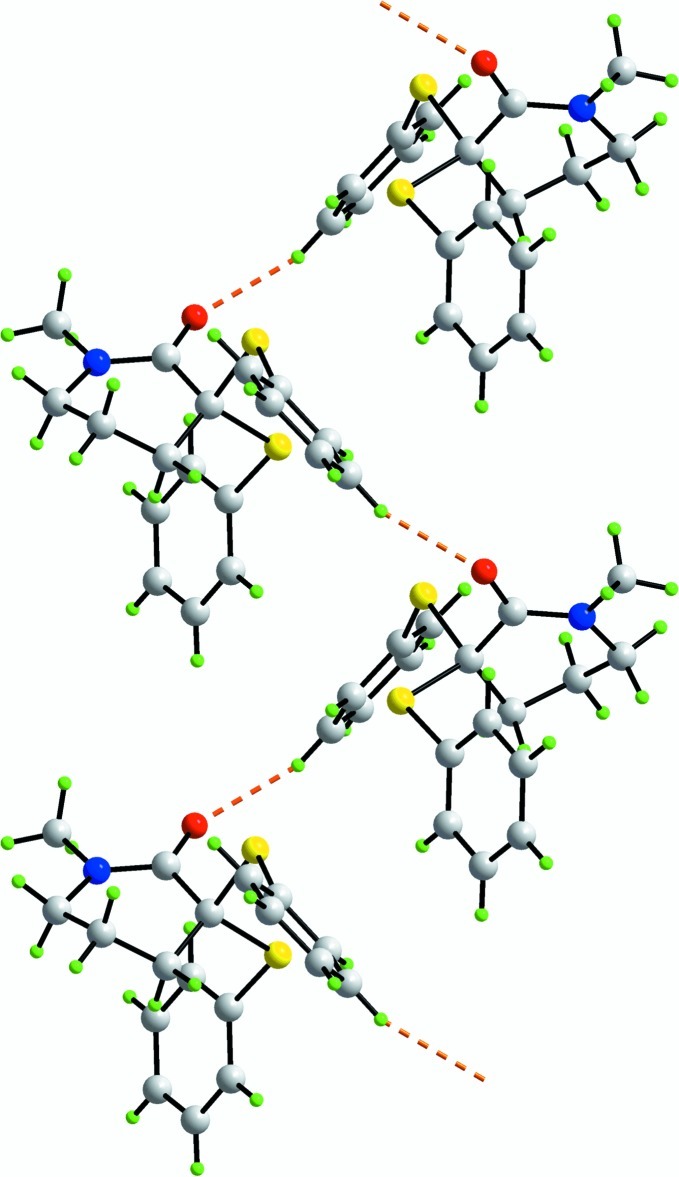
A view of the supramolecular chain in (I) mediated by C—H···O interactions, shown as orange dashed lines.

**Fig. 3.**
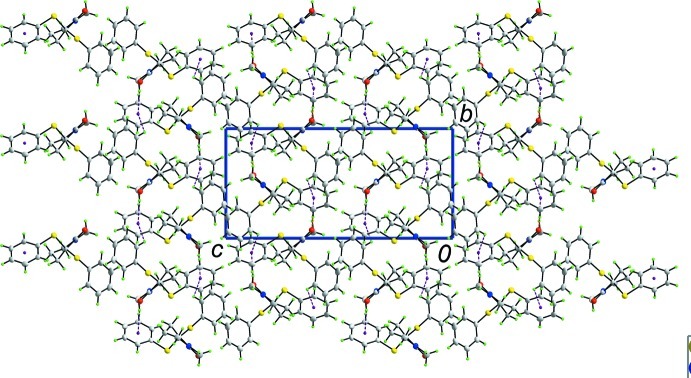
A view in projection down the a axis of the unit-cell contents for (I). The C—H···O and C—H···π interactions are shown as orange and purple dashed lines, respectively.

## supplementary crystallographic information

### Comment

As part of our on-going research on the conformational behaviour and electronic interactions in β-thio-carbonyl and β-bis-thio-carbonyl compounds, *e.g. N*-methoxy-*N*-methyl-2-[(4'-substituted) phenylthio]propanamides and 3,3-bis[(4'- substituted) phenylthio]-1-methyl-2-piperidones, using spectroscopic, theoretical and X-ray diffraction methods (Olivato *et al.*, 2009; Zukerman-Schpector *et al.* 2010, 2011, Vinhato *et al.*, 2011), the title compound, (I), was synthesized and its crystal structure determined.

In (I), Fig. 1, the piperidone ring is in a distorted half-chair conformation with the C4 atom lying 0.606 (2) Å out of the plane defined by the other five atoms (r.m.s. deviation = 0.1197 Å). The ring puckering parameters are: q_2_ = 0.4368 (19) Å, q_3_ = 0.2886 (18) Å, QT = 0.5235 (18) Å, φ_2_ = 216.7 (2) ° (Cremer & Pople, 1975). The S2-bound phenyl ring is almost perpendicular to the plane through the piperidone ring [dihedral angle = 75.97 (6) °] whereas the S1-bond phenyl ring makes dihedral angles of 44.21 (7) and 59.92 (6) ° with those through the piperidone and S2-bound phenyl rings, respectively.

The crystal packing of (I) is sustained by C—H···O and C—H···π interactions, Table 1. The C—H···O interactions lead to the formation of an helical supramolecular chain along the *b* axis, Fig. 2. These chains are consolidated into a three-dimensional architecture *via* C—H···π interactions with the S1-benzene accepting two C—H···π contacts, Fig. 3.

### Experimental

Firstly, 1-methyl-2-piperidinone (2.3 g, 20 mmol) was added drop-wise to a cooled (195 K) solution of hexamethylphosphoramide (HMPA) (3.6 ml, 20 mmol), diisopropylamine (2.8 ml, 20 mmol) and butyllithium (13.2 ml, 1,52 mol.*L*^-1^ hexane solution) in THF (60 ml). After 20 minutes, diphenyl disulfide (4.4 g, 20 mmol) dissolved in THF (20 ml) was added dropwise to the enolate solution (Zoretic & Soja, 1976). The solution was stirred for 4 h at 195 K, then water (100 ml) was added at room temperature and extraction with dichloromethane was performed. The organic layer was dried over anhydrous sodium sulfate. After evaporation of the solvent, a crude solid was obtained. Purification through flash chromatography with a solution of hexane and ethyl acetate in a 7:3 ratio give the pure product (1.4 g, yield = 21%). Suitable crystals for X-ray analysis were obtained by vapour diffusion of *n*-hexane into a chloroform solution of (I) held at 283 K; m.p. 405–406 K. IR (cm^-1^): ν(C=O) 1663. NMR (CDCl_3_, p.p.m.): δ 1.87–1.91 (2*H*, multiplet), 1.96–1.99 (2*H*, multiplet), 2.92 (3*H*, singlet), 3.14–3.17 (2*H*, triplet, J = 6.1 Hz), 7.32–7.37 (4*H*, multiplet, Aryl-H), 7.38–7.40 (2*H*, multiplet, Aryl-H), 7.63–7.65 (4*H*, multiplet, Aryl-H). Analysis found: C 65.49, H 5.91, N 4.17%. C_18_H_19_ONS_2_ requires: C 65.62, H 5.81, N 4.25%.

### Refinement

The H atoms were geometrically placed (C—H = 0.95–0.99 Å) and refined as riding with *U_iso_*(H) = 1.2–1.5*U_eq_*(C).

### Crystal data

**Table:**

C_18_H_19_NOS_2_	*F*(000) = 696
*M**_r_* = 329.48	*D*_x_ = 1.331 Mg m^−^^3^
Orthorhombic, *P*2_1_2_1_2_1_	Cu *K*α radiation, λ = 1.54184 Å
Hall symbol: P 2ac 2ab	Cell parameters from 3533 reflections
*a* = 8.2103 (1) Å	θ = 4.5–75.9°
*b* = 9.8329 (1) Å	µ = 2.93 mm^−^^1^
*c* = 20.3686 (2) Å	*T* = 100 K
*V* = 1644.38 (3) Å^3^	Prism, colourless
*Z* = 4	0.35 × 0.30 × 0.25 mm

### Data collection

**Table:**

Agilent SuperNova Dual (Cu at zero) diffractometer with an Atlas detector	2769 independent reflections
Radiation source: fine-focus sealed tube	2728 reflections with *I* > 2σ(*I*)
Graphite monochromator	*R*_int_ = 0.016
Detector resolution: 10.4041 pixels mm^-1^	θ_max_ = 76.2°, θ_min_ = 5.0°
ω scans	*h* = −8→10
Absorption correction: multi-scan (*CrysAlis PRO*; Agilent, 2010)	*k* = −11→12
*T*_min_ = 0.427, *T*_max_ = 0.585	*l* = −25→24
4452 measured reflections	

### Refinement

**Table:**

Refinement on *F*^2^	Secondary atom site location: difference Fourier map
Least-squares matrix: full	Hydrogen site location: inferred from neighbouring sites
*R*[*F*^2^ > 2σ(*F*^2^)] = 0.025	H-atom parameters constrained
*wR*(*F*^2^) = 0.067	*w* = 1/[σ^2^(*F*_o_^2^) + (0.0401*P*)^2^ + 0.3751*P*] where *P* = (*F*_o_^2^ + 2*F*_c_^2^)/3
*S* = 1.09	(Δ/σ)_max_ < 0.001
2769 reflections	Δρ_max_ = 0.19 e Å^−^^3^
200 parameters	Δρ_min_ = −0.32 e Å^−^^3^
0 restraints	Absolute structure: Flack (1983), 818 Friedel pairs
Primary atom site location: structure-invariant direct methods	Flack parameter: 0.024 (14)

### Special details

**Table:**

Geometry. All s.u.'s (except the s.u. in the dihedral angle between two l.s. planes) are estimated using the full covariance matrix. The cell s.u.'s are taken into account individually in the estimation of s.u.'s in distances, angles and torsion angles; correlations between s.u.'s in cell parameters are only used when they are defined by crystal symmetry. An approximate (isotropic) treatment of cell s.u.'s is used for estimating s.u.'s involving l.s. planes.
Refinement. Refinement of *F*^2^ against ALL reflections. The weighted *R*-factor *wR* and goodness of fit *S* are based on *F*^2^, conventional *R*-factors *R* are based on *F*, with *F* set to zero for negative *F*^2^. The threshold expression of *F*^2^ > σ(*F*^2^) is used only for calculating *R*-factors(gt) *etc*. and is not relevant to the choice of reflections for refinement. *R*-factors based on *F*^2^ are statistically about twice as large as those based on *F*, and *R*- factors based on ALL data will be even larger.

### Fractional atomic coordinates and isotropic or equivalent isotropic displacement parameters (Å^2^)

**Table:**

	*x*	*y*	*z*	*U*_iso_*/*U*_eq_	
S1	0.30204 (5)	1.02555 (4)	0.75801 (2)	0.01619 (10)	
S2	0.35543 (5)	0.81897 (4)	0.65673 (2)	0.01664 (10)	
N1	−0.07129 (19)	0.98484 (16)	0.66379 (7)	0.0177 (3)	
O1	0.15902 (17)	1.07356 (13)	0.62189 (6)	0.0212 (3)	
C1	0.0918 (2)	0.99466 (17)	0.66038 (8)	0.0152 (3)	
C2	0.1941 (2)	0.90147 (17)	0.70511 (8)	0.0134 (3)	
C3	0.0940 (2)	0.79738 (17)	0.74265 (8)	0.0146 (3)	
H3A	0.0652	0.7210	0.7132	0.018*	
H3B	0.1593	0.7606	0.7794	0.018*	
C4	−0.0604 (2)	0.86200 (18)	0.76924 (9)	0.0180 (4)	
H4A	−0.1205	0.7955	0.7965	0.022*	
H4B	−0.0325	0.9412	0.7970	0.022*	
C5	−0.1651 (2)	0.9070 (2)	0.71229 (9)	0.0216 (4)	
H5A	−0.2130	0.8260	0.6908	0.026*	
H5B	−0.2557	0.9639	0.7289	0.026*	
C6	−0.1655 (3)	1.0590 (2)	0.61425 (9)	0.0253 (4)	
H6A	−0.1307	1.0306	0.5703	0.038*	
H6B	−0.1470	1.1569	0.6194	0.038*	
H6C	−0.2816	1.0392	0.6199	0.038*	
C7	0.3549 (2)	0.93152 (17)	0.82931 (8)	0.0142 (3)	
C8	0.2861 (2)	0.96954 (18)	0.88918 (8)	0.0188 (3)	
H8	0.2052	1.0385	0.8906	0.023*	
C9	0.3364 (3)	0.90622 (19)	0.94685 (8)	0.0216 (4)	
H9	0.2906	0.9328	0.9877	0.026*	
C10	0.4531 (2)	0.80460 (19)	0.94479 (9)	0.0203 (4)	
H10	0.4880	0.7626	0.9844	0.024*	
C11	0.5195 (2)	0.76355 (18)	0.88520 (9)	0.0197 (4)	
H11	0.5975	0.6923	0.8839	0.024*	
C12	0.4711 (2)	0.82740 (18)	0.82761 (8)	0.0159 (3)	
H12	0.5170	0.8003	0.7868	0.019*	
C13	0.2397 (2)	0.71592 (18)	0.60188 (8)	0.0152 (3)	
C14	0.2444 (2)	0.57523 (19)	0.60717 (8)	0.0184 (4)	
H14	0.3047	0.5338	0.6416	0.022*	
C15	0.1608 (2)	0.49458 (19)	0.56209 (9)	0.0220 (4)	
H15	0.1653	0.3983	0.5655	0.026*	
C16	0.0713 (2)	0.5546 (2)	0.51225 (9)	0.0221 (4)	
H16	0.0131	0.4998	0.4818	0.027*	
C17	0.0671 (3)	0.6953 (2)	0.50700 (9)	0.0261 (4)	
H17	0.0059	0.7366	0.4728	0.031*	
C18	0.1512 (3)	0.77555 (19)	0.55114 (8)	0.0224 (4)	
H18	0.1486	0.8717	0.5469	0.027*	

### Atomic displacement parameters (Å^2^)

**Table:**

	*U*^11^	*U*^22^	*U*^33^	*U*^12^	*U*^13^	*U*^23^
S1	0.0193 (2)	0.01418 (17)	0.01510 (18)	−0.00298 (17)	−0.00493 (16)	0.00065 (14)
S2	0.01155 (18)	0.0246 (2)	0.01375 (18)	−0.00025 (17)	−0.00070 (15)	−0.00305 (16)
N1	0.0151 (7)	0.0212 (7)	0.0167 (6)	0.0036 (6)	−0.0037 (6)	0.0015 (6)
O1	0.0241 (7)	0.0212 (6)	0.0182 (6)	−0.0059 (6)	−0.0033 (5)	0.0061 (5)
C1	0.0182 (8)	0.0152 (8)	0.0122 (7)	−0.0006 (7)	−0.0022 (6)	−0.0011 (7)
C2	0.0126 (7)	0.0160 (7)	0.0116 (7)	−0.0014 (7)	−0.0022 (6)	0.0003 (6)
C3	0.0162 (8)	0.0145 (7)	0.0132 (7)	−0.0018 (7)	−0.0004 (6)	0.0001 (7)
C4	0.0171 (9)	0.0199 (8)	0.0171 (8)	−0.0015 (7)	0.0029 (7)	−0.0002 (7)
C5	0.0135 (8)	0.0262 (9)	0.0252 (9)	−0.0001 (8)	0.0015 (8)	−0.0002 (7)
C6	0.0243 (10)	0.0303 (9)	0.0213 (8)	0.0088 (9)	−0.0098 (8)	−0.0003 (8)
C7	0.0127 (7)	0.0163 (7)	0.0135 (7)	−0.0029 (7)	−0.0037 (7)	−0.0017 (6)
C8	0.0199 (9)	0.0186 (8)	0.0180 (8)	0.0014 (8)	−0.0014 (7)	−0.0038 (7)
C9	0.0284 (10)	0.0242 (9)	0.0123 (7)	−0.0039 (8)	−0.0003 (7)	−0.0025 (7)
C10	0.0236 (9)	0.0200 (8)	0.0173 (8)	−0.0066 (8)	−0.0079 (7)	0.0022 (7)
C11	0.0151 (8)	0.0170 (8)	0.0270 (9)	−0.0019 (7)	−0.0062 (8)	0.0011 (7)
C12	0.0123 (8)	0.0183 (8)	0.0172 (8)	−0.0015 (7)	0.0000 (6)	−0.0031 (7)
C13	0.0117 (8)	0.0229 (8)	0.0109 (7)	0.0010 (7)	0.0006 (6)	−0.0026 (6)
C14	0.0164 (8)	0.0235 (8)	0.0154 (7)	0.0058 (8)	0.0007 (7)	−0.0013 (7)
C15	0.0226 (9)	0.0221 (9)	0.0213 (8)	0.0008 (8)	0.0015 (8)	−0.0053 (7)
C16	0.0176 (8)	0.0317 (10)	0.0170 (8)	−0.0014 (8)	0.0006 (7)	−0.0088 (7)
C17	0.0266 (10)	0.0351 (10)	0.0167 (8)	0.0049 (9)	−0.0100 (8)	−0.0018 (8)
C18	0.0293 (10)	0.0223 (8)	0.0157 (8)	0.0014 (8)	−0.0045 (8)	0.0006 (7)

### Geometric parameters (Å, º)

**Table:**

S1—C7	1.7755 (17)	C7—C12	1.400 (3)
S1—C2	1.8535 (17)	C8—C9	1.392 (2)
S2—C13	1.7826 (17)	C8—H8	0.9500
S2—C2	1.8396 (18)	C9—C10	1.385 (3)
N1—C1	1.344 (2)	C9—H9	0.9500
N1—C6	1.466 (2)	C10—C11	1.390 (3)
N1—C5	1.468 (2)	C10—H10	0.9500
O1—C1	1.233 (2)	C11—C12	1.389 (2)
C1—C2	1.541 (2)	C11—H11	0.9500
C2—C3	1.519 (2)	C12—H12	0.9500
C3—C4	1.518 (2)	C13—C14	1.388 (3)
C3—H3A	0.9900	C13—C18	1.393 (2)
C3—H3B	0.9900	C14—C15	1.394 (3)
C4—C5	1.511 (3)	C14—H14	0.9500
C4—H4A	0.9900	C15—C16	1.385 (3)
C4—H4B	0.9900	C15—H15	0.9500
C5—H5A	0.9900	C16—C17	1.388 (3)
C5—H5B	0.9900	C16—H16	0.9500
C6—H6A	0.9800	C17—C18	1.381 (3)
C6—H6B	0.9800	C17—H17	0.9500
C6—H6C	0.9800	C18—H18	0.9500
C7—C8	1.395 (2)		
			
C7—S1—C2	104.46 (7)	H6B—C6—H6C	109.5
C13—S2—C2	101.69 (8)	C8—C7—C12	119.57 (16)
C1—N1—C6	117.01 (16)	C8—C7—S1	118.46 (13)
C1—N1—C5	126.51 (15)	C12—C7—S1	121.82 (13)
C6—N1—C5	116.48 (15)	C9—C8—C7	119.86 (17)
O1—C1—N1	121.59 (16)	C9—C8—H8	120.1
O1—C1—C2	120.39 (16)	C7—C8—H8	120.1
N1—C1—C2	118.00 (15)	C10—C9—C8	120.14 (17)
C3—C2—C1	113.80 (14)	C10—C9—H9	119.9
C3—C2—S2	111.23 (11)	C8—C9—H9	119.9
C1—C2—S2	109.75 (11)	C9—C10—C11	120.47 (17)
C3—C2—S1	114.18 (11)	C9—C10—H10	119.8
C1—C2—S1	102.29 (11)	C11—C10—H10	119.8
S2—C2—S1	104.91 (9)	C12—C11—C10	119.60 (17)
C4—C3—C2	110.44 (14)	C12—C11—H11	120.2
C4—C3—H3A	109.6	C10—C11—H11	120.2
C2—C3—H3A	109.6	C11—C12—C7	120.32 (16)
C4—C3—H3B	109.6	C11—C12—H12	119.8
C2—C3—H3B	109.6	C7—C12—H12	119.8
H3A—C3—H3B	108.1	C14—C13—C18	119.44 (16)
C5—C4—C3	108.91 (14)	C14—C13—S2	120.21 (14)
C5—C4—H4A	109.9	C18—C13—S2	120.25 (14)
C3—C4—H4A	109.9	C13—C14—C15	120.15 (17)
C5—C4—H4B	109.9	C13—C14—H14	119.9
C3—C4—H4B	109.9	C15—C14—H14	119.9
H4A—C4—H4B	108.3	C16—C15—C14	120.10 (18)
N1—C5—C4	111.75 (15)	C16—C15—H15	120.0
N1—C5—H5A	109.3	C14—C15—H15	120.0
C4—C5—H5A	109.3	C15—C16—C17	119.63 (18)
N1—C5—H5B	109.3	C15—C16—H16	120.2
C4—C5—H5B	109.3	C17—C16—H16	120.2
H5A—C5—H5B	107.9	C18—C17—C16	120.46 (19)
N1—C6—H6A	109.5	C18—C17—H17	119.8
N1—C6—H6B	109.5	C16—C17—H17	119.8
H6A—C6—H6B	109.5	C17—C18—C13	120.21 (18)
N1—C6—H6C	109.5	C17—C18—H18	119.9
H6A—C6—H6C	109.5	C13—C18—H18	119.9
			
C6—N1—C1—O1	7.5 (3)	C3—C4—C5—N1	−48.7 (2)
C5—N1—C1—O1	−172.54 (16)	C2—S1—C7—C8	−115.91 (15)
C6—N1—C1—C2	−171.12 (14)	C2—S1—C7—C12	68.49 (16)
C5—N1—C1—C2	8.9 (3)	C12—C7—C8—C9	1.6 (3)
O1—C1—C2—C3	−172.11 (15)	S1—C7—C8—C9	−174.07 (15)
N1—C1—C2—C3	6.5 (2)	C7—C8—C9—C10	−0.7 (3)
O1—C1—C2—S2	−46.74 (18)	C8—C9—C10—C11	−0.9 (3)
N1—C1—C2—S2	131.88 (15)	C9—C10—C11—C12	1.6 (3)
O1—C1—C2—S1	64.23 (17)	C10—C11—C12—C7	−0.6 (3)
N1—C1—C2—S1	−117.15 (15)	C8—C7—C12—C11	−1.0 (3)
C13—S2—C2—C3	61.95 (12)	S1—C7—C12—C11	174.61 (13)
C13—S2—C2—C1	−64.87 (12)	C2—S2—C13—C14	−111.92 (15)
C13—S2—C2—S1	−174.12 (8)	C2—S2—C13—C18	71.68 (16)
C7—S1—C2—C3	33.97 (14)	C18—C13—C14—C15	−0.1 (3)
C7—S1—C2—C1	157.38 (11)	S2—C13—C14—C15	−176.56 (14)
C7—S1—C2—S2	−88.05 (9)	C13—C14—C15—C16	−0.7 (3)
C1—C2—C3—C4	−42.33 (18)	C14—C15—C16—C17	0.8 (3)
S2—C2—C3—C4	−166.90 (11)	C15—C16—C17—C18	−0.1 (3)
S1—C2—C3—C4	74.61 (16)	C16—C17—C18—C13	−0.7 (3)
C2—C3—C4—C5	63.97 (18)	C14—C13—C18—C17	0.8 (3)
C1—N1—C5—C4	13.1 (3)	S2—C13—C18—C17	177.28 (16)
C6—N1—C5—C4	−166.90 (15)		

### Hydrogen-bond geometry (Å, º)

Please define Cg1

**Table:**

*D*—H···*A*	*D*—H	H···*A*	*D*···*A*	*D*—H···*A*
C11—H11···O1^i^	0.95	2.32	3.237 (3)	163
C6—H6b···*Cg*1^ii^	0.98	2.95	3.606 (2)	125
C14—H14···*Cg*1^iii^	0.95	2.96	3.544 (2)	121

Symmetry codes: (i) −*x*+1, *y*−1/2, −*z*+3/2; (ii) −*x*+1, *y*−1/2, −*z*+1/2; (iii) −*x*, *y*−1/2, −*z*+3/2.
